# Supplementary material for: Microcystis viridis NIES-102 Cyanobacteria Lectin (MVL) Interacts with SARS-CoV-2 Spike Protein Receptor Binding Domains (RBDs) via Protein–Protein Interaction
Source: Int J Mol Sci. 2024 Jun 18;25(12):6696. doi: 10.3390/ijms25126696 (PMC11203576; doi:10.3390/ijms25126696)
Supplement: Supplementary file 1 [file ijms-25-06696-s001.zip › ijms-3048377-supplementary.pdf]

Supplementary materials

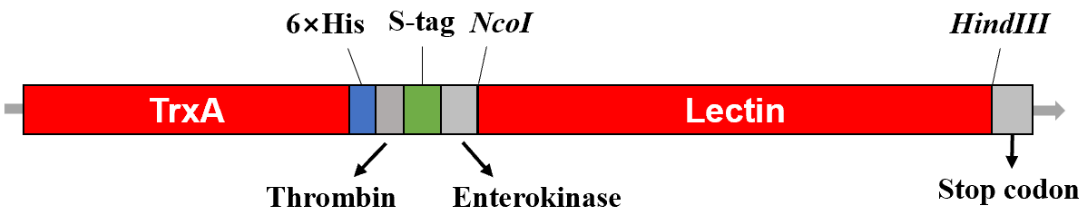

(a)

The ORF of pET32a-MVL
MSDKIIHLTDDSFDTDVLKADGAILVDFWAEWCGPCKMIAPILDEIADEYQGKLTVAK
LNIDQNPGTAPKYGIRGIPTLLLFKNGEVAATKVGALSKGQLKEFLDANLAGSGSGH
MHHHHHHSSGLVPRGSGMKETAAAKFERQHMDSPDLGTDDDDKAMASYKVNIPAGPLW
SNAEAQQVGPKIAAAHQGNFTGQWTTVVESAMSVVEVELQVENTGIHEFKTDVLAGPL
WSNDEAQKLGPQIAASYGAEFTGQWRTIVEGVMSVIQIKYTF*

(b)

Supplementary Fig. S1: Schematic diagram of an open reading frame (ORF) composed of lectin and thioredoxin (a) and amino acid sequence of TrxA-MVL fusion protein (b). The bolded portion represents the TrxA tag, and the underline section denotes the lectin sequence.

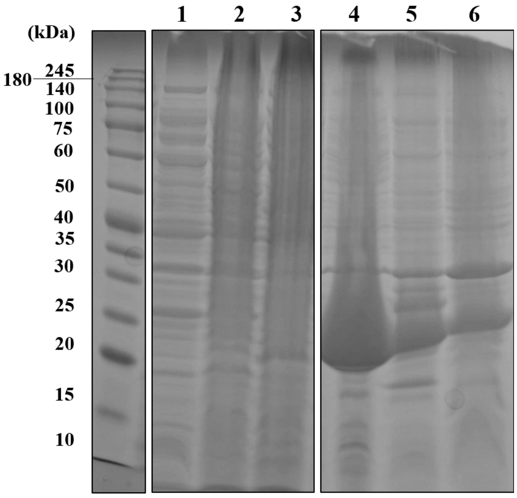

(a)

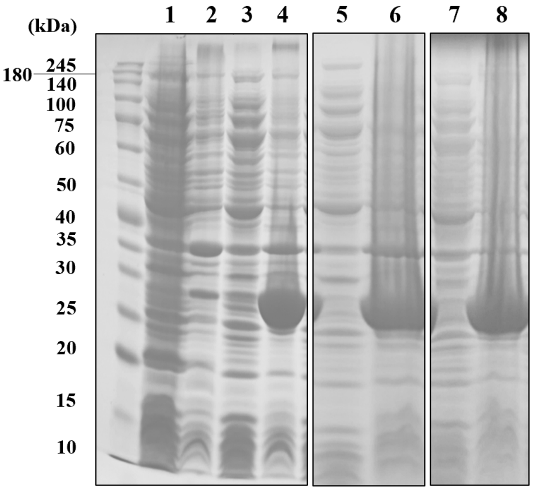

(b)

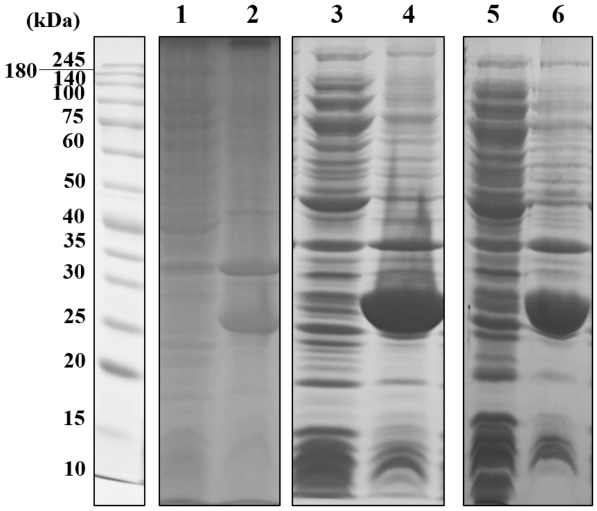

(c)

Supplementary Fig. S2: Optimization of culture conditions for MVL-TrxA expression

a: Host cell screening. Samples 1, 2, 3 correspond to the supernatant of BL21(DE3), Rosetta gami2 (DE3), BL21-CodonPlus (DE3)-RIPL; 4, 5, 6 correspond to the pellet of BL21(DE3), Rosetta gami2 (DE3), BL21-CodonPlus (DE3)-RIPL. **b**: Culture time screening. Samples 1 and 2 correspond to the supernatant and pellet after 4 h of culture of empty vector pET32a, 3 and 4 correspond to the supernatant and pellet after 4 h of cultivation, and 5 and 6 correspond to the supernatant and pellet after 8 h of cultivation, 7 and 8 correspond to the supernatant and pellet after 16 h of cultivation. **c**: Culture temperature screening. Samples 1 and 2 correspond to the supernatant and pellet after 4 h of cultivation at 37°C, 3 and 4 correspond to the supernatant and pellet after 4 h of cultivation at 23°C, and 5 and 6 correspond to the supernatant and pellet after 4 h of cultivation at 16°C.

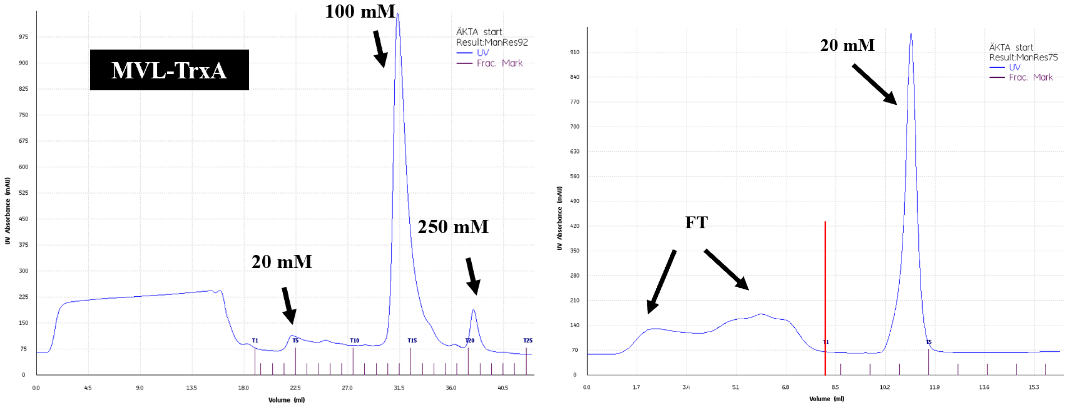

(a) (b)

Supplementary Fig. S3: The purification profiles of rMVL-Trx fusion protein (a) and thrombin-digested MVL-TrxA (b) on His-trap HP column.

Elution of His-tag protein was conducted by imidazole (20 mM, 100 mM and 250 mM). Flow throw fraction (FT in panel (b)) contained rMVL.

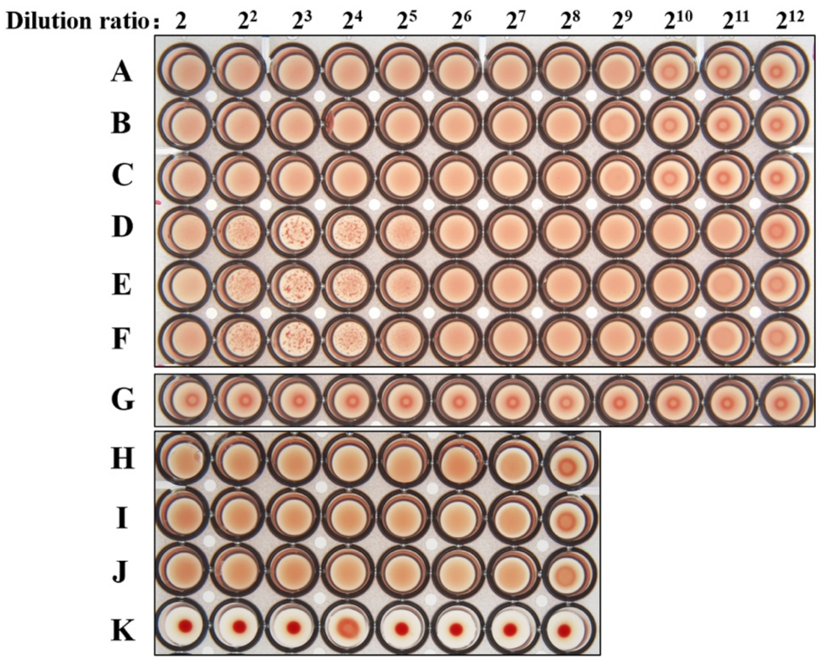

(a)

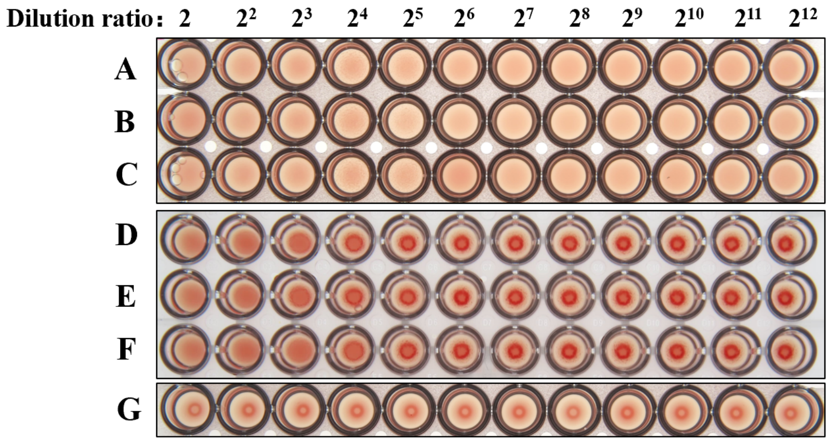

(b)

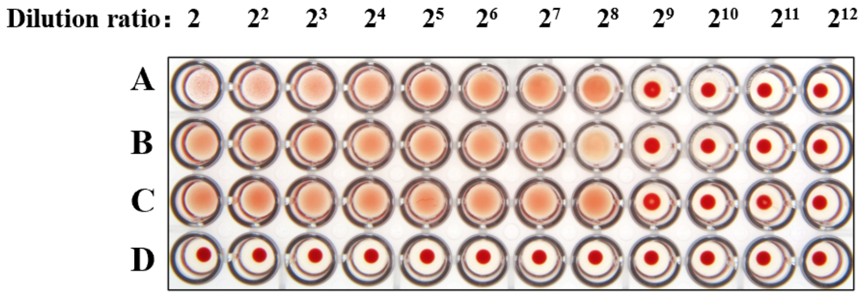

(c)

Supplementary Fig. S4: Hemagglutination assays of lectins using rabbit erythrocyte.

(a): Hemagglutination assay results of PHA-M (A-C), Jacalin (D-F) and Con A (H-G) lectins; negative controls (G, K). (b): Hemagglutination assay results of CSL3 (A-C) and DB1 (D-F) lectins; negative control (G). (c): Hemagglutination assay result of recombinant MVL (A-C); negative control (D).

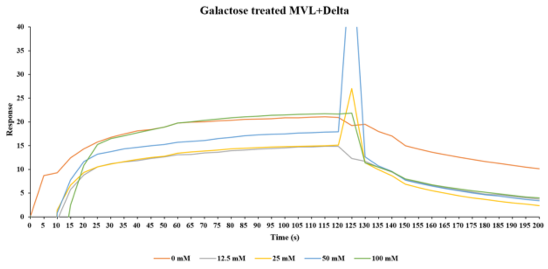

(a)

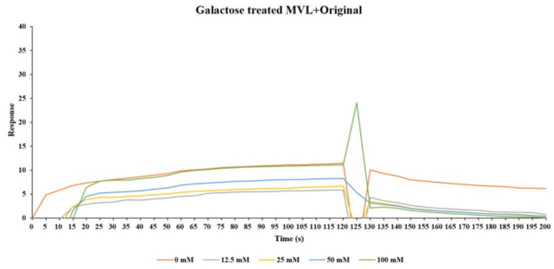

(b)

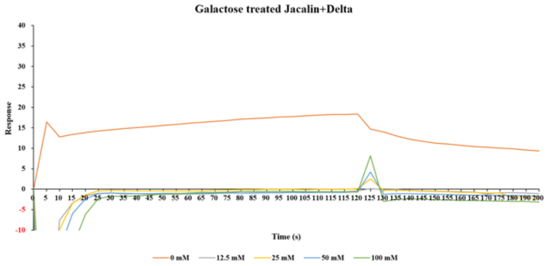

(c)

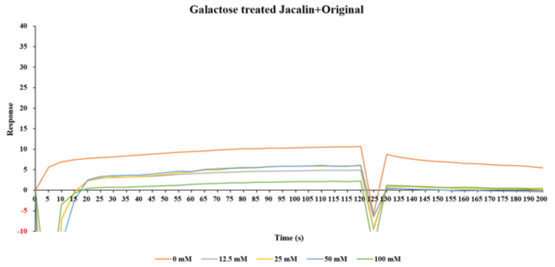

(d)

Supplementary Fig. S5: Interaction between lectins and Delta or original spike protein RBDs after galactose treatment. MVL (a, b) and Jacalin (c, d) were treated with galactose (0 mM, 12.5 mM, 25 mM, 50 mM, 100 mM) at room temperature for 30 min before SPR, which was performed as for Fig. 2

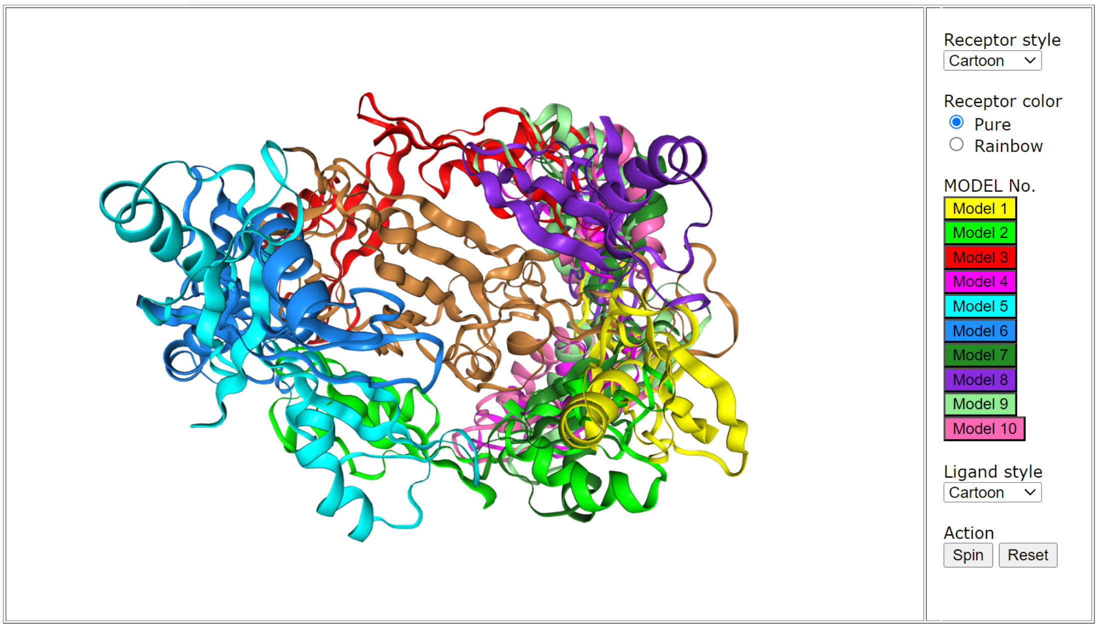

Complex Template Information (Click to Show)

Summary of the Top 10 Models

Rank	1	2	3	4	5	6	7	8	9	10
Docking Score	-293.37	-292.08	-278.35	-276.21	-274.59	-273.05	-272.92	-272.77	-272.52	-270.25
Confidence Score	0.9462	0.9449	0.9287	0.9258	0.9236	0.9214	0.9212	0.9210	0.9206	0.9172
Ligand rmsd (Å)	75.42	72.08	48.04	63.95	67.58	62.97	72.23	65.08	64.56	64.41
Interface residues	model_1	model_2	model_3	model_4	model_5	model_6	model_7	model_8	model_9	model_10

(a)

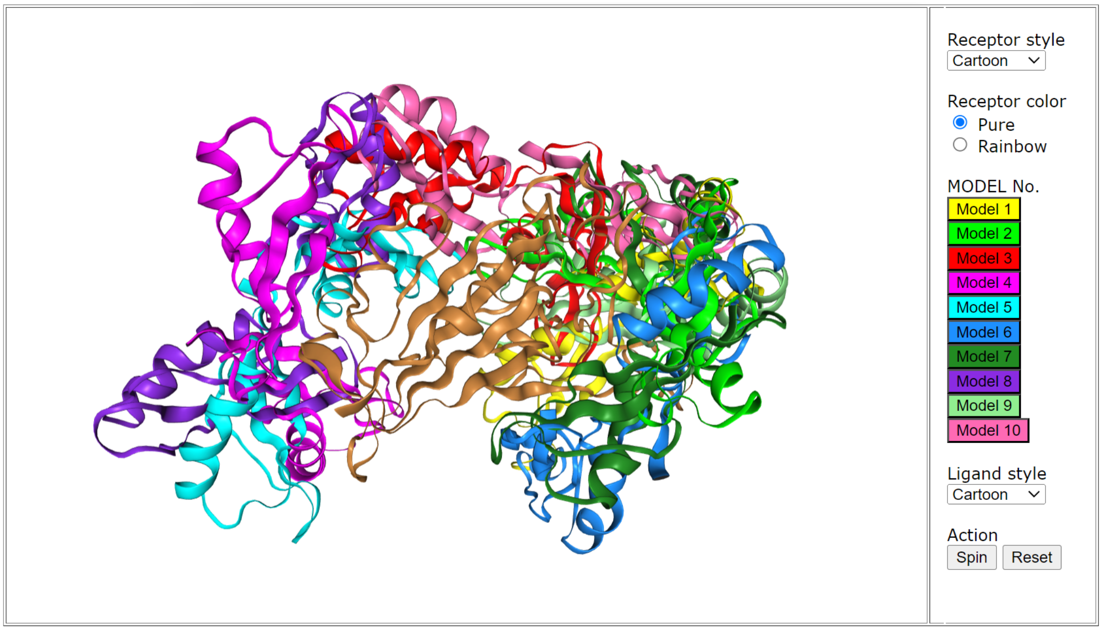

Complex Template Information (Click to Show)

Summary of the Top 10 Models

Rank	1	2	3	4	5	6	7	8	9	10
Docking Score	-296.37	-281.67	-279.67	-272.26	-265.95	-258.61	-256.60	-256.46	-255.62	-254.14
Confidence Score	0.9492	0.9330	0.9304	0.9202	0.9104	0.8977	0.8940	0.8937	0.8921	0.8892
Ligand rmsd (Å)	75.27	72.39	72.11	62.86	67.55	63.91	64.13	60.62	71.54	65.23
Interface residues	model_1	model_2	model_3	model_4	model_5	model_6	model_7	model_8	model_9	model_10

(b)

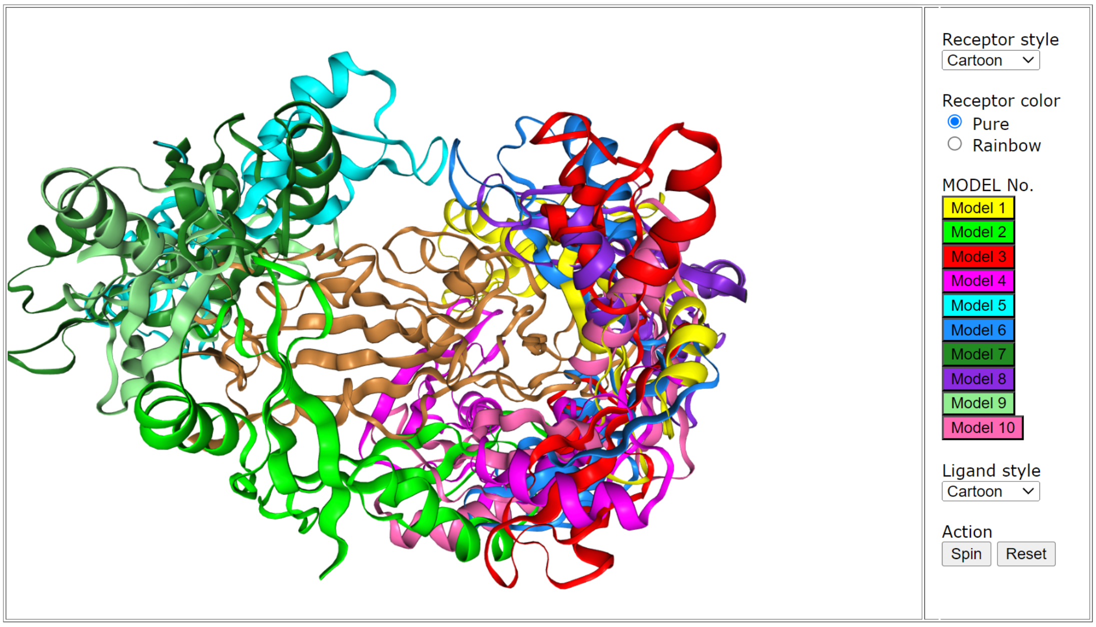

Complex Template Information (Click to Show)

Summary of the Top 10 Models

Rank	1	2	3	4	5	6	7	8	9	10
**Docking Score**	-294.01	-281.03	-278.36	-276.93	-273.31	-272.82	-269.07	-267.41	-265.60	-264.52
**Confidence Score**	0.9469	0.9322	0.9287	0.9268	0.9217	0.9210	0.9154	0.9128	0.9099	0.9081
**Ligand rmsd (Å)**	75.34	48.07	64.27	64.16	67.48	72.22	60.49	86.00	62.84	65.01
**Interface residues**	model_1	model_2	model_3	model_4	model_5	model_6	model_7	model_8	model_9	model_10

(c)

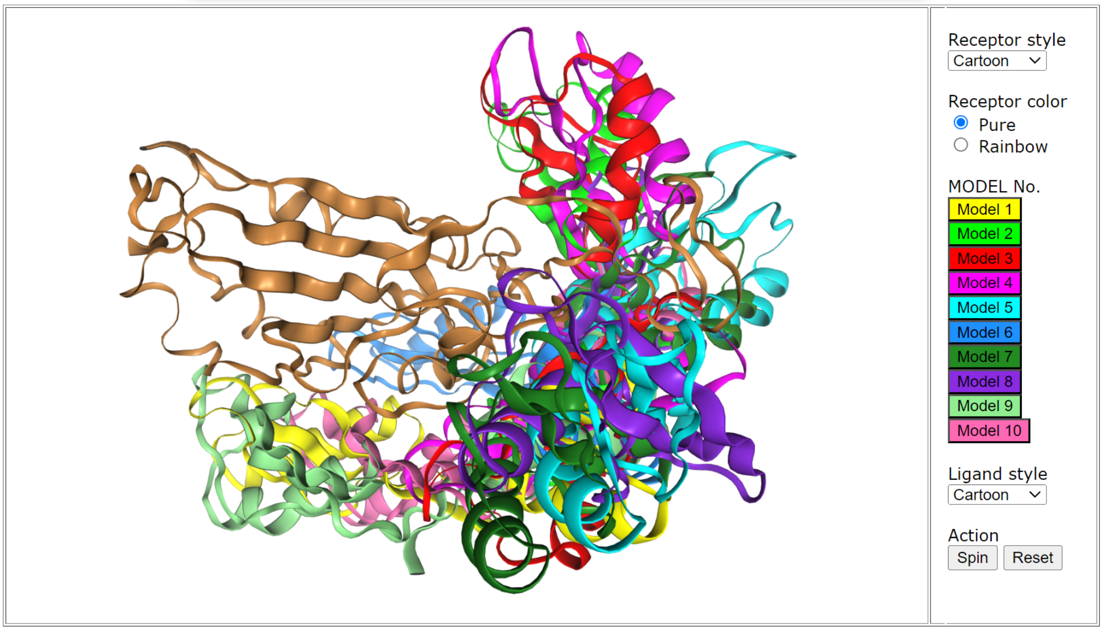

Complex Template Information (Click to Show)

Summary of the Top 10 Models

Rank	1	2	3	4	5	6	7	8	9	10
**Docking Score**	-307.74	-297.35	-289.80	-283.11	-282.17	-280.75	-279.02	-278.64	-277.12	-273.65
**Confidence Score**	0.9591	0.9501	0.9425	0.9348	0.9336	0.9318	0.9296	0.9291	0.9271	0.9222
**Ligand rmsd (Å)**	71.96	65.07	73.19	66.56	83.07	64.93	82.19	75.16	69.49	66.97
**Interface residues**	model_1	model_2	model_3	model_4	model_5	model_6	model_7	model_8	model_9	model_10

(d)

Supplementary Fig. S6: Docking simulation of MVL and spike protein RBDs variants.

The top 10 models of the complex interaction between MVL and spike protein RBDs variants, alpha (a), delta (b), omcrion (c), and original (d), were estimated by protein-protein docking simulation using the HDOCK server that based on hybrid docking strategies, template-based modeling and *ab initio* docking.

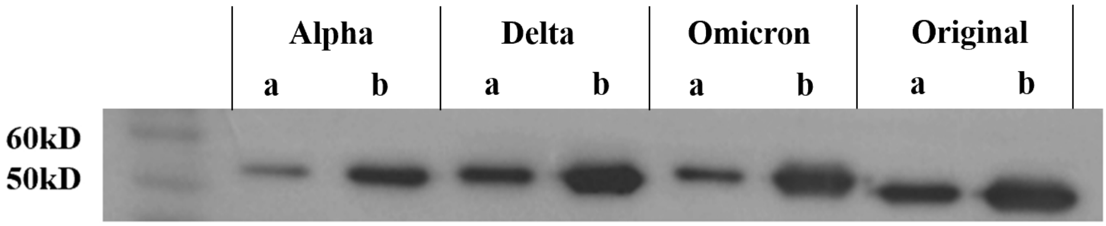

Supplementary Fig. S7. Western blotting profiles of recombinant SARS-CoV-2 spike protein RBDs (Alpha, Delta, Omicron and original) conjugated with sfGFP. Proteins purified by nickel column affinity chromatography were detected with anti-EGFP primary antibody. Lane a: 50 mM imidazole eluted fractions for each indicated RBD, lane b: 250 mM imidazole eluted fractions for each indicated RBD.
